# How Transparent and Reproducible Are Studies That Use Animal Models of Opioid Addiction?

**DOI:** 10.1111/adb.70027

**Published:** 2025-04-07

**Authors:** Justine C. Blackwell, Julia Beitner, Alex O. Holcombe

**Affiliations:** ^1^ School of Psychology University of Sydney Sydney Australia; ^2^ Department of Psychology Goethe University Frankfurt Frankfurt am Main Germany; ^3^ Clinical Psychology, Central Institute of Mental Health, Medical Faculty Mannheim University of Heidelberg Mannheim Germany; ^4^ Addiction Behavior and Addiction Medicine, Central Institute of Mental Health, Medical Faculty Mannheim University of Heidelberg Mannheim Germany; ^5^ Psychiatry and Psychotherapy, Central Institute of Mental Health, Medical Faculty Mannheim University of Heidelberg Mannheim Germany; ^6^ German Center for Mental Health (DZPG), Partner Site Mannheim‐Heidelberg‐Ulm Mannheim Germany

**Keywords:** addiction, animal models, bias minimization, opioids, reproducibility, translation, transparency

## Abstract

The reproducibility crisis in psychology has caused various fields to consider the reliability of their own findings. Many of the unfortunate aspects of research design that undermine reproducibility also threaten translation potential. In preclinical addiction research, the rates of translation have been disappointing. We tallied indices of transparency and accurate and thorough reporting in animal models of opioid addiction from 2019 to 2023. By examining the prevalence of these practices, we aimed to understand whether efforts to improve reproducibility are relevant to this field. For 255 articles, we report the prevalence of transparency measures such as preregistration, registered reports, open data and open code, as well as compliance to the Animal Research: Reporting of In Vivo Experiments (ARRIVE) guidelines. We also report rates of bias minimization practices (randomization, masking and data exclusion), sample size calculations and multiple corrections adjustments. Lastly, we estimated the accuracy of test statistic reporting using a version of *StatCheck*. All the transparency measures and the ARRIVE guideline items had low prevalence, including no cases of study preregistration and no cases where authors shared their analysis code. Similarly, the levels of bias minimization practices and sample size calculations were unsatisfactory. In contrast, adjustments for multiple comparisons were implemented in most articles (76.5%). Lastly, *p*‐value inconsistencies with test statistics were detected in about half of papers, and 11% contained statistical significance errors. We recommend that researchers, journal editors and others take steps to improve study reporting and to facilitate both replication and translation.

## Graphical abstract

This study assessed transparency, reproducibility and bias minimization practices in animal models of opioid addiction research from 2019 to 2023. Key indices included analysis script sharing, preregistration, masking and randomization, among others. Our findings reveal substantial room for improvement to enhance transparency, reproducibility and the translational potential of pre‐clinical addiction research.

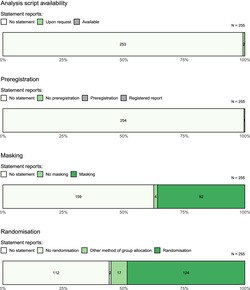

Confidence in psychological findings was shaken by poor rates of successful replications [1, 2] and researchers' reports of engagement in questionable research practices (QRPs) [3]. This ‘replication crisis’ has triggered discussion about how research is performed [2] and calls for more replication, including of addiction research [4].

Relatedly, disappointing levels of successful translation of preclinical research to clinical trials led some to declare a ‘translational research crisis’ [5]. The causes of the translational research crisis and the replication crisis overlap. Discussed as problems in both crises are researchers engaging in QRPs (see Table 1 for some common types) and a publication bias whereby journals favour articles that find statistical significance [10]. Moreover, unacceptably high risk of bias, in part due to failures in randomization and masking (or blinding), and inappropriate data exclusion likely play a role in both crises [14, 15].

**TABLE 1:** Questionable research practices contributing to the reproducibility crisis.

Questionable research practice	Definition	Implications	Consequences	Associated target variables^a^
HARKing	The researcher adjusts their a priori hypotheses after seeing results to more accurately ‘predict’ the study's outcome [6]	Exploratory research (‘we are not sure what is happening here so we will test for a few things to try and find out’) is misrepresented as confirmatory research (‘we think there is X effect here, we will test for it’)	The evidential weight for an effect is overestimated [6]	Preregistration Registered reports
*p*‐hacking	The undisclosed omission, transformation, combination of variables until statistical significance is reached [7]	By retesting the hypothesis multiple times, *p*‐hacking violates the assumptions of null hypothesis significance testing [8]	Validity of results undermined [8] Likelihood of finding a false positive increases [8]	Preregistration Registered reports Open data Open code
Outcome switching	Swapping the variables of interest in a study after seeing the results, often to reach significance [9] May occur consciously or unconsciously [10]	Misrepresents efficacy of a treatment at preclinical or clinical stages [9]	Mislead future clinical research [9] Precludes clinicians from making fully informed decisions about preclinical treatment efficacy [11, 12] Increased risk of finding a false positive [8] Not considering totality of results [10]	Preregistration Registered reports Open data Open code
Selective reporting	Omitting variables after observing the results, often in order to achieve significant results [9] Can also be referred to as underreporting when analyses, experiments or subjects are omitted [13]	Misrepresents efficacy of a treatment at preclinical or clinical stages [9] Data aggregation efforts (meta‐analyses, systematic reviews) cannot include all data generated for a certain outcome [9]	Precludes clinicians from making fully informed decisions about preclinical treatment efficacy [11, 12] Mislead future clinical research [9]	Preregistration Registered reports Open data Open code

^a^ The prevalence of these variables are being examined in this study. They may help detect the presence of the questionable research practice or mitigate its impact.

Procedures encouraging transparency and reproducibility in research, which hope to improve rigour and reduce bias in turn, may help [10]. Literatures with high rates of transparent practices facilitate reproducibility and allow increased confidence in results [16]. The current study aims to understand the extent to which the animal models of opioid addiction (AMOA) literature already employs such practices and the room that remains for improvement. The research practices examined in the current study are briefly outlined.

The sharing of raw data and analysis scripts can improve transparency and reproducibility. This practice facilitates scrutiny to encourage improvement and self‐correction and allows corroboration of claims, together increasing the credibility of results [17, 18]. Despite the benefits, data and analysis script sharing in addiction research appears to be rare [9, 19, 20].

Preregistration is widely considered to be part of the solution to the replication and translational crises [10, 13, 16, 21]. Preregistration involves uploading a study's hypotheses, research design and statistical analyses to an online repository prior to the collection of data. Doing so facilitates the detection of deliberate or unintentional *p*‐hacking and QRPs as well as other benefits [8, 16].

The registered report format is an article format that can reduce publication bias; it involves a journal accepting a paper based on the motivation and methodology before the data have been collected [22]. We will assess the uptake of this practice in AMOA, which has not yet been widely examined in studies of animals, as well as the prevalence of preregistration.

Improving reporting standards is one goal of the Animal Research: Reporting of In Vivo Experiment (ARRIVE) guidelines. Including the methodological details ARRIVE prescribes allows readers to scrutinize studies more deeply while also improving the ability of future researchers to replicate the work [23]. Thus, improving reporting may encourage rigorous and transparent research, leading to more robust findings, better reproducibility and higher translation success rates [14, 24].

The ARRIVE guidelines state that it is ‘essential’ to specify whether masking was used or not for each step of an experiment to reduce the chance of researcher bias influencing a study's outcome [23], which could misdirect future work, including translation [5, 25].

Randomizing group allocation, which is also included in the ARRIVE Essential 10, reduces the risk of selection bias in experiments and evenly disperses known and unknown confounders between groups [26]. Without it, the inferential statistics typically used for hypothesis tests are invalid [23].

A literature built on studies with high statistical power is less likely to have false positives and inflated effect sizes [22]. When combined with publication bias, low power is associated with a decline in efficacy from the preclinical to the clinical stage [25]. Studies have found that most papers in many fields are underpowered, including preclinical neuroscience, preclinical stroke and preclinical multiple sclerosis [22, 25, 27]. Despite this, sample size calculations remain uncommon in preclinical research [28, 29].

Exclusion of outliers is a common practice. Although there are legitimate reasons to exclude outliers, what is considered outlying is not standardized in many fields [30]. This presents an opportunity for bias, as researchers may favour outlier definitions that find a significant result [8]. Reporting whether or not outliers were excluded and what criterion was used is preferable to readers not knowing if or how this was done, as it may affect how they interpret the results.

In addition to the above indices of transparency and bias minimization, this study examined two additional aspects that can affect the reliability of a study's results: multiple comparisons adjustment (MCA) and the accuracy of reported statistical tests.

Statistical analysis often involves running multiple tests for a single hypothesis. Doing so introduces the problem of multiplicity: With each additional test, there is a higher chance of finding a false positive. The use of MCA can be particularly important for research leading to treatment development or policy change, as there is a greater cost of discovering a false positive than a false negative.

Full reporting of statistics, for example, following American Psychological Association guidelines, requires reporting the test type, the degrees of freedom, the test statistic and the *p* value. Each combination of test type, degrees of freedom and test statistic has only one valid *p* value. However, a substantial proportion of published psychology papers report impossible combinations, indicating at least one error in calculation or reporting [31]. If the *p* value is incorrect, the evidential value of the result is misrepresented.

To facilitate the detection of test statistic inconsistency, Epskamp and Nuijten [32] developed *StatCheck*. This R package recomputes *p* values from the reported tests and degrees of freedom and compares them to the published ones. Using *StatCheck,* Nuijten et al. [31] scanned more than 30 000 psychology papers and found decision errors—that is, inaccuracies that would change the significance of the statistical test at an alpha of 0.05—in 13%. We will use *StatCheck Simple Edition* to estimate the rate of test statistic inconsistencies in AMOA [33].

Poor reporting of quality research practices may mean they were not employed, risking increased bias. Moreover, a consumer of science should not have to trust that a certain practice was implemented; it should be clearly stated. We aim to ascertain whether efforts to improve the field's reproducibility are warranted by assessing the degree to which measures promoting transparency, accuracy and bias minimization reporting are already being implemented.

## 1 Methods

This is a retrospective observational study that appears to be the first of its kind in the animal addiction field. The aim was to estimate the prevalence of the variables discussed in the introduction and detailed in Table 2. The methods and planned analyses were preregistered (https://osf.io/q2z4d/). This manuscript is based on an honours thesis (https://ses.library.usyd.edu.au/handle/2123/32259).

**TABLE 2:** Study characteristics assessed in the current study.

Study characteristic	Response options	ARRIVE 2.0 guideline (where applicable)	Search terms and any additional instructions
Original or replication	Original		Read abstract *Replicat*
	Replication		
	Unsure		
Preregistration	No statement of preregistration	19. Provide a statement indicating whether a protocol (including the research question, key design features and analysis plan) was prepared before the study, and if and where this protocol was registered	*Regist, osf, aspredicted, preclinicaltrials*
	Statement of preregistration with link		
	Statement of preregistration but no link		
	There is a statement of non‐preregistration		
	This paper is a registered report		
Data availability	No statement regarding data availability	20. Provide a statement describing if and where study data are available	*Availab, request, reposit, data*
	Statement that some raw data are available via link		
	Statement some data available but link broken		
	Statement some data available but link absent		
	Unavailable—statement that the data are unavailable		
	Upon request		
Code availability	No code or syntax for analysis available		*Code, syntax, script*
	Syntax/code provided		
	Upon request^a^		
ARRIVE	Statement of compliance with ARRIVE or ARRIVE checklist in supplementary materials		*Arrive, guide, accordance, protocol, reporting*
	No mention of ARRIVE or compliance with another set of reporting guidelines		
	Other—mention of compliance with another set of guidelines		
Masking	Statement that there was (at least some) blinding	5. Describe who was aware of the group allocation at the different stages of the experiment (during the allocation, the conduct of the experiment, the outcome assessment and the data analysis)	*Blind, mask*
	Blinding not mentioned in relation to this study		
	Statement of no blinding/masking		
Randomization	Statement of some randomization Other method of group allocation given No allocation method mentioned Statement of NO randomization	4a. State whether randomization was used to allocate experimental units to control and treatment groups 4b. Describe the strategy used to minimize potential confounders such as the order of treatments and measurements, or animal/cage location. If confounders were not controlled, state this explicitly	*Random, alloc, assign*
Sample size justification	No justification given	2b. Explain how the sample size was decided. Provide details of any a priori sample size calculation, if done *If you have used an* a priori *sample size calculation, report* *the analysis method (*e.g.*, two‐tailed Student's t‐test with a 0.05 significance threshold)* *the effect size of interest and a justification explaining why an effect size of that magnitude is relevant* *the estimate of variability used (*e.g.*, standard deviation) and how it was estimated* *the power selected*	Read section ‘subjects’ or ‘animals’ in methods *Power, plan, priori*
	Power analysis/sample size planning		
	Past research		
	Practical constraints		
Multiple corrections	Corrected	7a. Provide details of the statistical methods used for each analysis, including software used *Relevant information to describe the statistical methods include the outcome measures; the independent variables of interest; the nuisance variables taken into account in each statistical test (*e.g., *as blocking factors or covariates); what statistical analyses were performed and references for the methods used; how missing values were handled; adjustment for multiple comparisons the software package and version used, including computer code if available*	*Correct, bonf, holm, scheffe, tukey, Benj, family, FDR, false*
	No mention of correction method		
Exclusion	No statement of animal exclusion	3b. For each experimental group, report any animals, experimental units, or data points not included in the analysis and explain why. If there were no exclusions, state so	*Exclu, outl, discard, sacrif*
	Animals were excluded from the study		
	Statement of no animal exclusion		
Exclusion reasons	Outlier exclusion		
	Other		
	Outlier exclusion and other reason(s)		
	No reason given		
	Not applicable (no exclusion mentioned)		
Supplementary files^b^	Yes		*Supplementary, supporting, appendi*
	No		
	Yes, but link absent/broken		

*Note:* The ARRIVE 2.0 guidelines text is taken from The ARRIVE guidelines 2.0: Updated guidelines for reporting animal research [23]. Text in italics in this column is taken from the elaborated guidelines in Reporting animal research: Explanation and elaboration for the ARRIVE guidelines 2.0 [24].

^a^ Unlike ‘data availability’, there were so few instances of analysis code being shared in our pilot coding that we did not feel it necessary to expand the response options beyond the original three options.

^b^ Serves to remind coders to check supplementary files. Not a variable of interest.

### 1.1 Sample

**FIGURE 1:**
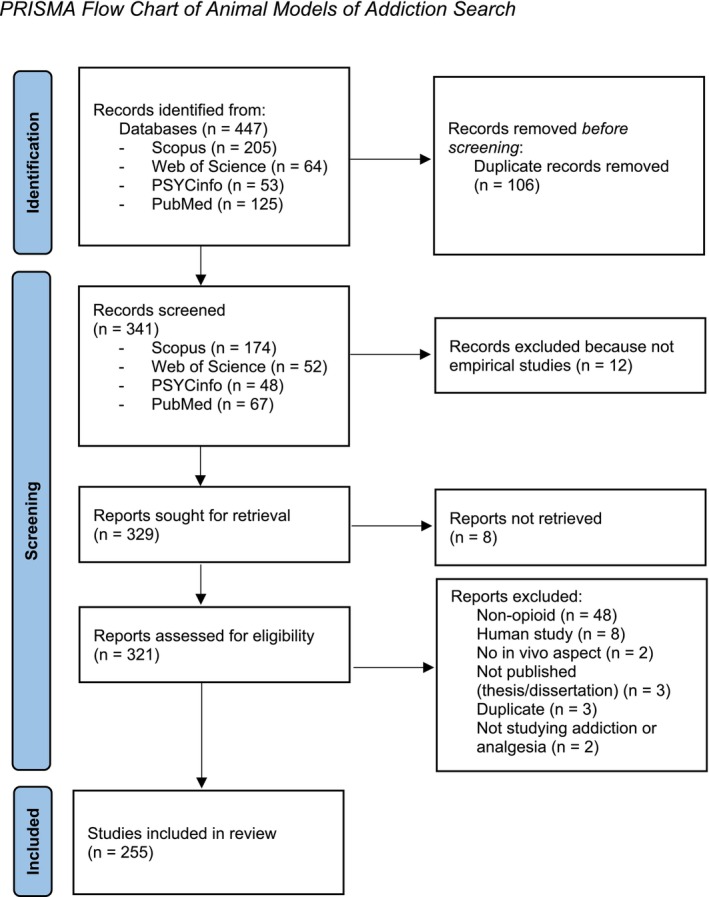
PRISMA flow chart of animal models of addiction search.

Our sampling process and exclusions can be seen in Figure 1. We used the following search string to find AMOA articles:addict* OR substance abuse OR drug addiction OR drug treatment AND opioid OR opiate OR heroin AND treatment OR treat* AND behavior* OR behaviour*.

We searched Scopus, Web of Science, PsycINFO and PubMed. We limited results to ‘article’, ‘empirical study’ and ‘animal’, written in English and published between 2019 and 2023. In Scopus, the results were also limited to relevant research areas (neuroscience, psychology, pharmacology, toxicology, pharmaceutics and multidisciplinary).

For a study to be included, it had to satisfy the following criteria: Be an empirical study; include an in vivo animal study; include administration of opioids; and study the effects of opioids or an opioid addiction treatment. The exclusion criterion was that the article had not been published in a journal.

We initially expected to take a random sample of the AMOA literature. However, upon completing the search, we found that the entire number of papers (*N* = 255) was feasible to study.

There were 50 articles published in 2019; 62 in 2020 and 2021; 57 in 2022; and 24 in 2023. Table 3 presents the journals that contained four or more articles in our sample and whether they mandate the ARRIVE guidelines and provide the registered report format. A list of all journals can be found at https://osf.io/gkn74.

**TABLE 3:** Most frequent journals in our dataset.

Journal name	Number of articles	Per cent	ARRIVE endorsement	Adopted registered reports
*Addiction Biology*	19	7.45	N^a^	N^b^
*Neuropharmacology*	18	7.06	E	N
*Psychopharmacology*	11	4.31	N	N
*Pharmacology, Biochemistry and Behavior*	11	4.31	E	N
*Neuropsychopharmacology*	11	4.31	N	N^b^
*Behavioural Brain Research*	10	3.92	E	N
*Frontiers in Pharmacology*	9	3.53	N^a^	N^b^
*Neuroscience Letters*	8	3.14	E	N
*Drug and Alcohol Dependence*	7	2.75	E	Y
*Molecular Psychiatry*	6	2.35	N^a^	N^b^
*International Journal of Molecular Sciences*	6	2.35	M	N
*Behavioural Pharmacology*	6	2.35	N	N
*Progress in Neuro‐Psychopharmacology and Biological Psychiatry*	5	1.96	E	N
*Journal of Psychopharmacology*	5	1.96	M	N^b^
*Journal of Pharmacology and Experimental Therapeutics*	5	1.96	N	N
*Frontiers in Molecular Neuroscience*	5	1.96	N^a^	N^b^
*Pain*	4	1.57	N	N
*Journal of Neuroscience*	4	1.57	N	N
*International Journal of Neuropsychopharmacology*	4	1.57	E	N
*Frontiers in Behavioral Neuroscience*	4	1.57	N^a^	N^b^
*Acta Pharmacologica Sinica*	4	1.57	M	N^b^
Total articles	162	63.5%		

*Note:* Only journals with four or more articles were included in this table (*N* = 21). For the full list of journals (*N* = 93), see Table S4. Three of the top 21 journals mandate use of the ARRIVE guidelines, whereas seven more encourage it. One of the top 21 journals supports the registered report format. ARRIVE endorsement status was derived from the ARRIVE website (arriveguidelines.org/supporters/journals) and from the journals' webpages and guides to authors. Registered report adoption is taken from the Centre for Open Science: Registered Reports website (cos.io/initiatives/registered‐reports) and individual journals' web pages. This information was current as of 12 March 2024.

Abbreviations: E = ARRIVE guidelines encouraged by the journal, M = ARRIVE guidelines mandated by the journal, N = journal does not specifically mention ARRIVE guidelines or Registered Report acceptance on website.

^a^ Journal publisher has a policy encouraging use of the ARRIVE guidelines, but there is no specific mention in relation to the journal.

^b^ Journal publisher states acceptance of registered report format, but there is no specific mention of registered reports in relation to the journal.

### 1.2 Pilot Coding

All articles were coded by two coders. Coders used a Google sheet codebook (https://osf.io/chvgw) developed by the four coders throughout the pilot coding process. After included variables had been finalized, Coder 1 completed the first round of pilot coding on five articles. After Coder 1 had ensured functionality of the codebook, Coder 1, Coder 2 and Coder 3 coded a small selection of studies together and further adjusted the codebook. Next, all four coders coded five articles. Coding proper commenced when all four coders agreed on the responses and were satisfied that the search terms were effective.

All pilot coding articles were selected from a search of preclinical addiction literature not specific to opioids. This meant there was no overlap in the articles used for pilot coding and those included in the final selection.

### 1.3 Coding Procedure

The coding instructions (https://osf.io/ah54e) were developed to standardize our coding procedure. In essence, each variable's relevant search terms were looked for in the article and supplementary materials using the search function. Some variables also required scanning relevant parts of the article. For example, to assess whether the paper included a justification of sample size, the ‘subjects’ or ‘animals’ paragraph was read, and search terms were looked for.

Between 51 and 91 articles were allocated to each Coder 2, Coder 3 and Coder 4, and all articles were double coded by Coder 1. 90.6% of articles were coded by two people. An additional 23 papers were not double coded due to the time constraints of one coder. However, given the high level of interrater agreement (see Table S3), we do not consider this a major limitation [34].

Importantly, coders were instructed to code generously—that is, we wanted to be biased in the direction of a high estimate of papers following best practices. This was in recognition of the fact that our measures are imperfect, and we may risk systematically underestimating the prevalence of some practices by not including a relevant search term. By coding in a manner that gives the benefit of the doubt, we hope to partly compensate for any threats to the accuracy of our estimates in this respect and therefore not unfairly censure the AMOA field.

The operationalization of randomization in this study was broad and was not confined to randomized group allocation. Whereas randomly assigning animals to groups is essential for minimizing bias and avoiding confounders, so too is randomizing treatment order or housing location, for example [24, 29]. For these reasons and to keep to the principle of coding generously, the implementation of randomization of any study aspect was coded as satisfying our randomization variable. Further, to acknowledge alternative solutions to potential confounders, we searched studies for other allocation techniques.

Where supplementary files were available, these were checked for all characteristics but were not scanned for statistics to enter into *StatCheck Simple Edition* [33]. A variable for the presence or absence of supplementary files was created to remind the coders to check it, but this was not a variable of interest.

### 1.4 Statistical Analyses

Our aim was largely descriptive rather than to test hypotheses; we were interested in the proportions of articles that satisfied each target variable. Unless otherwise specified, the denominator is the total sample size (255). Additionally, we compared our results to those of previous research. Comparisons are made with studies who operationalized their variables in the same way as the present study. Where comparison results were taken from the study of a population of papers, we drew comparisons without the need for statistics as our study also measured the entire population of papers returned in our search that matched our inclusion criteria. Where comparison studies used samples, we compare our results using a two‐sample proportions *z*‐test [35, 36]. This calculation tests for significant differences between two proportions. All *z*‐tests were two‐tailed. We used the results from previous studies as benchmarks, meaning we did not account for the uncertainty of their estimates.

Analysis code and raw data are available at https://osf.io/q2z4d/.

## 2 Results

Our final sample consisted of 255 articles published between 2019 and 2023. After they were initially coded, discrepancies between coders were resolved; see Reconciling Coding Discrepancies (https://osf.io/fvjme). Essentially, responses that estimated the upper bound of prevalence rates were favoured (to give the benefit of the doubt), and the opinion of the most experienced coder resolved ambiguous cases. The average percentage agreement among all coders was 93.6% (range: 87.5%–99.54%) (Table S3).

### 2.1 Replication and Transparency

**FIGURE 2:**
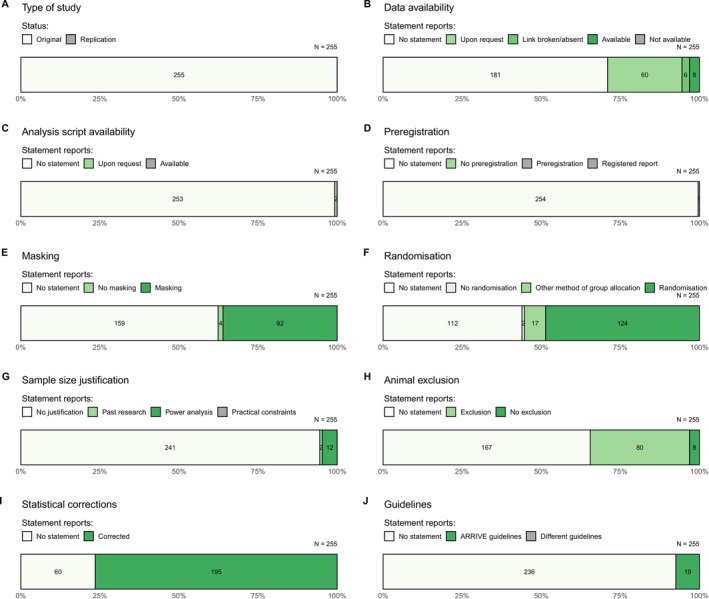
The prevalence of various characteristics for the 255 articles studied.

Overall, replication and transparency practices were uncommon (Figure 2). Tabulated results and comparisons to previous studies' estimates can be found in Table 4 (for full results, see Table S5).

**TABLE 4:** Results comparing the current study's findings to previous findings of transparency and replication practices.

Study characteristic	Our result			Previous study (field)	Total *N* of previous study
	*N*	%			
Replications	0	0%	0.4% [0.00072, 0.023]	Adewumi et al. [19] (addiction medicine)^a^	244
			0.2% [0.0017, 0.0023]	Lee [37] (psychology)	84 834
			0% [0, 0.037]	Norris et al. [20] (smoking addiction)	100
Data availability					
States available and accessible	8	3.1%	2%^c^	Hamilton et al. [17] (health and medicine meta‐research)^a^	2 121 580
			8.2% [0.054, 0.12]	Adewumi et al. [19] (addiction medicine)^a^	244
			7% [0.034, 0.14]	Norris et al. [20] (smoking addiction)	100
			7% [0.04, 0.12]	Hardwicke et al. [38] (psychology)	156
States available but inaccessible	6	2.4%^b^	5.1% [0.026, 0.098]	Hardwicke et al. [38] (psychology)	156
Upon request	60	23.5%	9% [0.048, 0.16]	Norris et al. [20] (smoking addiction)	100
			1.3% [0.0035, 0.046]	Hardwicke et al. [38] (psychology)	156
States not available	0	0%	2.05% [0.0088, 0.047]	Adewumi et al. [19] (addiction medicine)^a^	244
			1% [0.0018, 0.054]	Norris et al. [20] (smoking addiction)	100
			0.6% [0.0011, 0.035]	Hardwicke et al. [38] (psychology)	156
No statement	181	71%	87% [0.82, 0.91]	Adewumi et al. [19] (addiction medicine)^a^	244
			92%^c^	Hamilton et al. [17] (health and medicine meta‐research)^a^	2 121 580
			80.8% [0.74, 0.86]	Hardwicke et al. [38] (psychology)	156
			84% [0.76, 0.9]	Norris et al. [20] (smoking addiction)	100
Code availability					
States available (and accessible)	0	0%	0.8% [0.0023, 0.029]	Adewumi et al. [19] (addiction medicine)^a^	244
			1% [0.0018, 0.054]	Norris et al. [20] (smoking addiction)	100

		0.1%^c^	Hamilton et al. [17] (health and medicine meta‐research)^a^	

		1.3% [0.0035, 0.046]	Hardwicke et al. [38] (psychology)	156
No statement	253	99.2%	98.7%[0.95, 1]	Hardwicke et al. [38] (psychology)	156
			99% [0.95, 1]	Norris et al. [20] (smoking addiction)	100
			99.2% [0.97, 1]	Adewumi et al. [19] (addiction medicine)^a^	244
			99.9%^c^	Hamilton et al. [17] (health and medicine meta‐research)^a^	2 121 580

*Note:* The total number of articles that we assessed in the current study was 255. The confidence intervals are Wilson score intervals calculated by the Proportion Confidence Interval Calculator using the pwr library in R (Statskingdom, https://www.statskingdom.com/proportion‐confidence‐interval‐calculator.html).

Abbreviation: CI = confidence intervals.

^a^ This study examined human and animal studies. See Table S1 for more information on the previous studies presented here.

^b^ This percentage combines our response options of ‘states available but link broken’ and ‘states available but link absent’ to make comparisons to previous studies appropriate.

^c^ As the total number of included studies in this analysis is unclear given the article, we were unable to compute confidence intervals.

None (0%) of the articles described replication as a primary goal in the abstract, meaning all papers reviewed were original empirical research. Comparison of this finding to previous estimates of the proportion of replication studies in addiction medicine ([19], 0.4%, *Z* = 0.63, *p* = 0.529; [20], 0%, *Z* = 0, *p* = 1) and psychology ([39], 0.2%, Z = 0.45, *p* = 0.653) did not reveal statistically significant differences.

The raw data were available and accessible for only 3.1% of articles. This result was similar to that found in a previous study of health and medicine human and animal studies [17] and not significantly different from estimates of addiction medicine papers ([19], 8.2%, *Z* = 1.56, *p* = 0.110; [20], 7%, *Z* = 1.26, *p* = 0.208). However, we found a higher rate of data ‘available upon request’ (23.5%) than was found in studies of smoking addiction randomized controlled trials (RCTs) ([20], 9%, *Z* = ‐2.79, *p* = 0.005) and psychology ([38], 1.3%, *Z* = ‐4.78, *p* < 0.001). The proportion of studies with no statement regarding data availability was lower in our study (71%) compared to Hamilton et al.'s [17] review (92%) and significantly lower than estimates from addiction medicine ([19], 87%, *Z* = 2.79, *p* = 0.005; [20], 84%, *Z* = 2.22, *p* = 0.026) and psychology ([38], 80.8%, *Z* = 1.64, *p* = 0.101).

None (0%) of the articles we investigated shared their analytical code. This was comparable to that found by a study of health and medicine meta‐research ([17], 0.1%) and was not significantly different from estimates of addiction medicine ([19], 0.8%, *Z* = 0.9, *p* = 0.368; [20], 1%, *Z* = 1, *p* = 0.317) and psychology research ([38], 1.3%, *Z* = 1.14, *p* = 0.254).

### 2.2 Bias Minimization Practices

Table 5 displays the results presented below and compares our findings to results from previous preclinical studies.

**TABLE 5:** Results comparing the current study's findings to previous findings of bias minimization practices.

Study characteristic	Our result			Previous study (field)	Total *N* of previous study
	*N*	%			
Preregistration					
Statement of preregistration and preregistration accessible	0	0%	0% [0, 0.024]	Hardwicke et al. [38] (psychology)	156
			2.9% [0.017, 0.063]	Adewumi et al. [19] (addiction medicine)^a^	244
			72% [0.63, 0.8]	Norris et al. [20] (smoking addiction)	100
Statement of no preregistration	1	0.4%	0% [0, 0.015]	Adewumi et al. [19] (addiction medicine)^a^	244
			0% [0, 0.024]	Hardwicke et al. [38] (psychology)	156
No statement regarding preregistration	254	99.6%	97.1% [0.94, 0.99]	Adewumi et al. [19] (addiction medicine)^a^	244
			100% [0.98, 1]	Hardwicke et al. [38] (psychology)	156
Registered reports	0	0%			
Masking (any mention, including statement of no masking implemented)	96^b^	37.6%^b^	12.3% [0.12, 0.13]	Menke et al. [29] (biomedical)^c^	51 312
			34.5% [0.29, 0.41]	Leung et al. [28] (animal welfare, analgesia or anaesthesia)^c^, ^d^	236
Randomization (any mention)	124	48.6%	36.3% [0.36, 0.37]	Menke et al. [29] (biomedical)^c^	51 312
			17.1% [0.085, 0.31]	Ting et al. [40] (rheumatology)^c^	41
Sample size calculation^e^	12	4.7%	29% [0.26, 0.33]	Fergusson et al. [14] (pain and anaesthesiology)^c^, ^d^	604
			12.8% [0.1, 0.16]	Kousholt et al. [41] (Danish)^c^, ^d^	500
			9.7% [0.066, 0.14]	Leung et al. [28] (animal welfare, analgesia or anaesthesia)^c^, ^d^	236
			7.3% [0.071, 0.075]	Menke et al. [29] (biomedical)^c^	51 312
			< 1% [0.0055, 0.018]	Vesterinen et al. [27] (multiple sclerosis)^c^	1117
			0.7% [0.0046, 0.011]	Macleod et al. [42] (biomedical disease models)^c^	2671
			0% [0, 0.086]	Ting et al. [40] (rheumatology)^c^	41
Data exclusion (any mention)	80	31.4%	38.4% [0.34, 0.43]	Kousholt et al. [41] (Danish)^c^, ^d^	500
			19.5% [0.1, 0.34]	Ting et al. [40] (rheumatology)^c^	41

*Note:* The total number of articles that we assessed in the current study was 255. The confidence intervals are Wilson score intervals calculated by the Proportion Confidence Interval Calculator using the pwr library in R (Statskingdom, https://www.statskingdom.com/proportion‐confidence‐interval‐calculator.html).

Abbreviation: CI = confidence intervals.

^a^ This study examined human and animal studies. See Table S2 for more information on the previous studies presented here.

^b^ This number combines the results from the current study for response options ‘Yes, masking mentioned in relation to this study’ and ‘Statement of no masking mentioned in relation to this study’ to make comparisons to previous studies appropriate.

^c^ This study examined only animal studies. See Table S2 for more information on the previous studies presented here.

^d^ This study compared results from two time periods. The previous study's result presented here is the most recent time period studied. See Supplementary Table S2 for results from both time periods of interest.

^e^ This variable reflects the proportion of studies that used power analyses or other sample size planning calculations used. This variable was measured in our study as the ‘power analysis/sample size planning’ response option of ‘sample size justification’.

No (0%) papers stated that any of their studies were preregistered or were in a registered report format. The former result was not significantly different to a review of addiction medicine papers ([19], 2.9%, *Z* = 1.72, *p* = 0.085) and psychology literatures ([38], 0%, *Z* = 0, *p* = 1), but it is significantly less than found for clinical studies of smoking addiction ([20], 72%, *Z* = 10.61, *p* < 0.001). Just one paper (0.4%) explicitly stated that their research was not preregistered.

Masking was mentioned in the methods of 36.1% of papers, and 1.6% of papers included a statement that no masking was used. These numbers, combined to allow comparison to previous findings (37.7%), are slightly higher than the finding from Leung et al.'s [28] review of the animal welfare, analgesia and anaesthesia literature (34.5%) and larger than that found in a more general study of the biomedical literature ([29], 12.3%).

48.6% of papers mentioned the use of randomization of some kind at some point in the experiment. This is a larger percentage than that found in the biomedical literature by Menke et al. [29] and in the rheumatology literature by Ting et al. [40]. Additionally, we found reference to alternative group allocation methods in 6.7% of papers and a statement of no randomization in 0.8% of papers.

94.5% of articles did not include a justification for their sample size, whereas 0.8% cited previous research as their justification. 4.7% justified their sample size with a sample size calculation or a power analysis; comparable results from previous studies varied widely (range 0%–29%; see Table 5). Of the 12 papers that used sample size calculations to determine their sample sizes (Table S7), 0% of these reported the effect size type used (e.g., Cohen's *d*). Additionally, 9 did not include the effect size magnitude used in the calculation, and 1 was unclear, leaving only 2 papers that reported the effect size magnitude used.

3.1% of papers included statements of no exclusion of data or animals. 31.4% of papers did report excluding data, which was a smaller proportion than reported in a study of Danish literature ([41], 38.4%) but larger than a study of rheumatology ([40], 19.5%). Of this 31.4% (80 articles), outlying data were the sole cause for exclusion in 9 papers. A further 7 made exclusions because of outlying data and an additional reason. One paper gave no reason, and the remaining 63 papers cited reasons other than outlying data.

7.5% of articles stated that they complied with the ARRIVE reporting guidelines. No other guidelines for reporting of preclinical in vivo experiments were cited, meaning 92.5% of articles did not appear to follow any reporting guidelines.

76.5% of the articles examined reported using some form of multiple comparison adjustment (which could be certain statistical tests, such as Tukey's HSD), which was greater than found for cardiovascular RCTs and human pain research ([43], 45%; [44], 28.3%) (Table 6).

**TABLE 6:** Multiple comparisons adjustments: Current study's findings and previous findings.

Study characteristic	Our result (number articles)	Our result (%)	Comparative study	Comparative result
Multiple comparisons corrections present	195	76.5%	Khan et al. [44]^a^	28.3%
			Gewandter et al. [43]^b^	45%
No mention of multiple comparisons correction	60	23.5%		
Total	255	100%		

*Note:* As with the current study, the papers presented in this table coded the entire population; thus, null hypothesis significance testing is not appropriate.

^a^ Khan et al. [44] examined the rate of multiple corrections adjustment in 511 articles (published between 2015 and 2018) containing multiplicity in human cardiovascular randomized control trials.

^b^ Gewandter et al. [43] looked at the rate of adjustment for multiple corrections in the primary analyses of 161 randomized control trials from human fields studying non‐invasive pharmacological treatments or interventional treatments for pain, published between 2006 and 2012.

Lastly*, StatCheck* detected at least one test statistic in 76.5% of papers reviewed in this study (Table 7). Of these 195 papers, 95 (48.7%) contained a *p*‐value error that did not affect statistical significance, which is almost identical to the proportion found by Nuijten et al. [31] in a study of eight psychology journals. Similarly, 24 papers out of the 195 (12.3%) contained apparent *p*‐value errors that changed whether the result was statistically significant, which, again, was very close to Nuijten et al.'s [31] result of 12.9%.

**TABLE 7:** Test statistic accuracy.

Study characteristic	Our result	Nuijten et al. [31]
Test statistic detection rate	76.5%	54.4%
Percentage of papers containing a non‐decision error	48.7%^a^	49.6%
Percentage of papers containing a decision error	12.3%^a^	12.9%

*Note:* As with the current study, the paper by Nuijten et al. [31] examined a population; thus, null hypothesis significance testing is not appropriate. Nuijten et al. [31] investigated the accuracy of reported test statistics in 16 695 articles from eight ‘flagship’ psychology journals from 1985 to 2013 in which null hypothesis significance testing results were detected.

^a^ This percentage includes only those papers where test statistics were detected. In our study, *N* = 195 (of a possible 255). In Nuijten et al.' study [31], *N* = 16 695 (of a possible 30 717).

## 3 Discussion

Reproducibility in preclinical research, and potential for successful translation, is supported by transparent, accurate reporting and rigorous methods [10, 14]. Here, we manually reviewed papers studying opioid use and opioid alternatives to assess transparency, compliance with ARRIVE guidelines and statistical errors.

Zero papers stated that replications were one of their main goals, which, unfortunately, is in line with estimates from addiction and psychology literatures. As in those fields, replications may not be done in AMOA due to pressures of working in a competitive field that rewards novelty over replications [8, 21]. Alternatively, researchers may be replicating foundational effects before building on them but, due to publishing space limitations, may be unable to publish them (or they may be published as part of a larger study but not have been highlighted as a goal). However, given our findings of low reporting of bias minimization and transparency practices (discussed below), the absence of reported replications is concerning. This suggests that calls for replication attempts in human addiction research are relevant to AMOA [4].

More data availability statements including ‘available upon request’ statements in our population compared to related fields may reveal a relatively good awareness of data sharing as a practice and/or the considerable number of journals publishing AMOA research that require a data availability statement. This awareness may be unsurprising given the familiarity preclinical researchers are likely to have with data repositories; as a multidisciplinary field, these researchers have often been exposed to practices in an adjacent field such as proteomics where data sharing is common [45]. On the other hand, for only a small percentage of studies was the data accessible by our coders. Furthermore, Hardwicke and Ioannidis [18] revealed the inadequacy of the ‘available upon request’ solution when they were unable to retrieve data from authors for 68% of studies.

None of the articles we assessed appeared to have made their analysis code available, which unfortunately is similar to reports from other fields. This implies that obstacles to sharing code reported by previous studies may be relevant to AMOA: insufficient time, funding, or know‐how, plus concerns about potential misuse or misinterpretation of code and the associated data [46]. The currently low rates of data and code sharing prevent identification of possible computational errors in AMOA research, which have the potential to hinder translation to clinical settings. Gomes et al. [46] believe barriers to code and data sharing can be overcome with appropriate training, incentivized by funders and publishers.

None of the articles we studied were preregistered. In contrast, a clinical study of RCTs of smoking treatments found 72% were preregistered [20]. Thus, despite sometimes working alongside clinical trials, which top international medical journals will not publish unless they are preregistered [47], AMOA studies are rarely preregistered. Our result was similar to Adewumi et al.'s [19] result, which came from assessing a wider range of study types in addiction medicine research.

The recent creation of the animal‐specific study registries such as Preclinical Trials (https://preclinicaltrials.eu/) and Animal Study Registry (https://www.animalstudyregistry.org) addresses concerns that an absence of such was one reason for the low rates of preclinical preregistration [13, 40]. However, a lack of awareness about these sites or preregistration in general and the reasons for it may remain an obstacle for AMOA researchers, as in other fields [20]. This is likely also a reason that none of the articles in the population were registered reports, as well as the fact that few of the journals that publish most AMOA research offer registered reports (Table 3).

The AMOA literature would likely benefit from greater use of preregistration and registered reports, as they prompt researchers to consider bias minimization techniques and power analyses. Spitzer and Mueller [48] found that initial concerns about preregistration, such as reduced flexibility in data analysis and risks of intellectual property theft, were largely unsupported by actual experiences. Additionally, though preregistration takes time, this time primarily reflects a reallocation of effort from later to earlier stages in the research workflow [48].

An absence of data sharing and preregistration prevents interested parties from assessing research non‐publication and underreporting of animal subjects in AMOA. This hinders decision‐making at clinical stages [13].

AMOA experiments may also be influenced by bias as suggested by the low rates of mention of masking. Of the articles we studied, 1.6% included statements indicating that masking was *not* done in the study, which at least removes ambiguity. An analysis of preclinical researchers' attitudes towards masking revealed a lack of proficiency and doubt about the value or relevance, which are clear obstacles to adoption [49].

Nearly half of the articles we studied reported randomizing some aspect of their study or studies. This is higher than what has been found in some fields. However, it is unclear why use of randomization should not be higher, given that randomization procedures are ‘well established’ [5, 50]. Bebarta et al. [26] suggest the practice may be considered unnecessary by some animal researchers because of the higher homogeneity of animals compared to humans. Although there are times when randomization may not be appropriate or possible, appropriate reporting and defence of such decisions seem in order.

Very few articles provided a justification of their sample size. The incidence was similar to one related research area [28] but was substantially less than the field of pain and anaesthesiology [14]. This highlights the heterogeneity of fields adopting such practices. Importantly, none of the studies detailing sample size calculations provided enough detail to redo the calculation. The low prevalence of sample size calculations suggests that AMOA research may be underpowered, like other preclinical fields [22, 25, 27]. Although sample size calculations may not always be straightforward, compulsory reporting of how sample sizes are decided would encourage discussion between researchers about related difficulties and potential solutions.

A third of the papers reported that some data were excluded from analyses, and an additional 3.1% included a statement that no exclusion occurred. This suggests that the remaining 64% of papers are ambiguous regarding what exclusions were done (if any)—research that does not mention data exclusion does not always mean no data were excluded [13]. Of the papers that reported that they excluded data, 20% reported outlier exclusion as the reason. Inconsistent definitions of an outlier heighten the need for transparent reporting. As no articles were preregistered, we cannot know if researchers protected against the potential for biased data removal by deciding on exclusion criteria a priori. Regarding the papers without exclusion statements, the limited sharing of data and code would make unreported data exclusion hard to detect.

Of the few articles that claimed to comply with ARRIVE, only one reported on all four of the ‘Essential’ aspects that we coded (masking, randomization, sample size calculation and data exclusion) (see Table S6). Although not an exhaustive evaluation of ARRIVE adherence, this indicates that authors stating compliance with reporting guidelines is not enough for actual compliance. Hair et al. [51] similarly found that requesting completion of the ARRIVE guidelines was not enough to see adherence. Other research has suggested that reviewers must proactively enforce ARRIVE adherence to see significant improvement [52, 53, 54].

The three‐quarters of papers reporting adjustments for multiple comparisons was higher than found previously for clinical research [43, 44]. It should be noted, however, that many clinical trials are preregistered, typically with a primary outcome designated, lessening or eliminating the need for an adjustment. Nevertheless, this result is encouraging, and raising the rate of MCA may require minimal encouragement from reviewers and journals.

Our findings regarding inconsistency of statistics reported were similar to previous estimates by Nuijten et al. [31] in psychology. Given the importance of statistical significance in evaluating preclinical research for further investigation, even 12% of papers with a decision error is arguably unacceptable. Moreover, given the low rates of data sharing in AMOA, correcting these inconsistencies would be difficult [31]. We recommend that journals institute pre‐publication reporting consistency checks [55].

### 3.1 Solutions

A middle ground between enforcing adoption of practices and waiting for voluntary adoption is to enforce *reporting* of implementation or non‐implementation of crucial practices, as recommended by ARRIVE. Encouraging awareness in this way may accelerate voluntary change [23]. The ARRIVE guidelines attempt to improve understanding of transparent reporting with their informative ‘Explanation’ segment, available as an article or on the ARRIVE website (https://arriveguidelines.org/arrive‐guidelines; [24]). Furthermore, online tools are available to help plan rigorous research (Experimental Design Assistant, https://nc3rs.org.uk/our‐portfolio/experimental‐design‐assistant‐eda).

Whereas requiring use of ARRIVE may seem simple, experiences at journals indicate otherwise [40, 51, 56]. As such, the involvement of not just journal editors but also researchers, funders and universities (in short, all stakeholders) in the academic ecosystem is important [5]. Robson et al. [57] suggest a holistic approach and list a variety of actions each of these stakeholders can take to foster transparent research practices. Any solution that rests solely on one party may not be sustainable.

### 3.2 Limitations

Although we were able to study all relevant papers returned by our literature search, our search may nevertheless have missed a substantial number of papers. In addition, our generous coding style may have led to overestimations. This was done deliberately so that our research is not perceived as unfair criticism of the field and instead serves to motivate AMOA researchers to improve these rates.

Further, *StatCheck* makes mistakes. It misses many statistics because it depends on reporting statistics in APA format, but it can also have false positives. For example, it may misinterpret a chi‐square report because of the variety of glyphs that researchers and publishers use to depict the chi and the squared symbols [58]. Nevertheless, it has been found that implementing *StatCheck* during peer review reduces errors [59], and future versions of it or others' tools should do even better.

## 4 Conclusion

This is the first metascience study that we are aware of that focused on animal models of addiction. Our results, for our narrow domain of opioid‐related research, should prompt concern about the reproducibility of the AMOA literature. Moving forward, a variety of reforms and practices could be considered by AMOA researchers for improving transparency and reporting in the field.

## Ethics Statement

This research was deemed exempt from ethical review by the Research Integrity & Ethics Administration of the University of Sydney because it ‘(a) is negligible risk research (as defined in paragraph 2.1.7); and (b) involves the use of existing collections of data or records that contain only non‐identifiable data about human beings’ (National Statement 5.1.22).

## Conflicts of Interest

The authors declare no conflicts of interest.

## Supporting information


**Table S1.** Prevalence of transparency and replication practices as assessed by previous work.
**Table S2.** Prevalence of bias minimization practices in animal research as assessed by previous work.
**Table S3.** Percentage agreement between coders.
**Table S4.** List of all journals in our dataset.
**Table S5.** Prevalence of transparency and replication measures from the current study.
**Table S6.** Results of bias minimization variables from the current study.
**Table S7.** Results breakdown for articles including sample size calculations.
**Table S8.** Results Breakdown for Articles Including Mention of data exclusion.

## Data Availability

The raw data and analysis code are available at https://osf.io/q2z4d/.
