# Supplementary material for: How Transparent and Reproducible Are Studies That Use Animal Models of Opioid Addiction?
Source: Addict Biol. 2025 Apr 7;30(4):e70027. doi: 10.1111/adb.70027 (PMC11973454; doi:10.1111/adb.70027)
Supplement: Supplementary file 1 — Table S1. Prevalence of transparency and replication practices as assessed by previous work. Table S2. Prevalence of bias minimization practices in animal research as assessed by previous work. Table S3. Percentage agreement between coders. Table S4. List of all journals in our dataset. Table S5. Prevalence of transparency and replication measures from the current study. Table S6. Results of bias minimization variables from the current study. Table S7. Results breakdown for articles including sample size calculations. Table S8. Results Breakdown for Articles Including Mention of data exclusion. [file ADB-30-e70027-s001.docx]

**Supplementary Table 1**

*Prevalence of Transparency and Replication Practices as Assessed by Previous Work*

|  |  |  | Study | | | | |
| --- | --- | --- | --- | --- | --- | --- | --- |
| Study Characteristic | | Adewumi et al. (2021) | Norris et al. (2021) | Hamilton et al. (2023) | Hardwicke et al. (2020) | Makel et al. (2012) | Pui Yu Lee et al. (2022) |
| Field |  | Addiction medicine | Addiction (Smoking) | Health and medicine meta-research | Social sciences | Psychology | Psychology |
| Human or animal or both |  | Both^a^ | Human | Both | Human, neither^d^ | Unclear | Unclear |
| Publishing years of papers reviewed |  | 2014-2018 | 2018-2019 | 1781-2022 (interquartile range 2012-2018) | 2014-2017 | 1900-2012 | 2010-2021 |
| Total number of papers |  | 244 | 100 | 105 meta-analyses (2 121 580 articles) | 156 | 500 | 84 834 |
| Replications |  | .4% | 0% |  |  | 1.1% | 0.2% |
| Data availability (%) | States available (and accessible). | 11.5% (8.2%) | 7% | 8% (2%) ^b^ | 7% |  |  |
|  | States available but inaccessible. |  |  |  | 5.1% |  |  |
|  | Upon request. |  | 9% |  | 1.3% |  |  |
|  | States not available. | 2.05% | 1% |  | 0.6% |  |  |
|  | No statement. | 87% | 84% | 92% ^b^ | 80.8% |  |  |
| Code availability (%) | States available. | 0.8% | 1% | 0.5% ^c^ | 1.3% |  |  |
|  | No statement. | 99.2% | 99% | 99.5% | 98.7% |  |  |

*Note*. The first four rows describe characteristics of the previous works. The following rows present their results. RCT: Randomized Control Trial.

^a^The distribution of studies was 225 human and 19 animal

^b^This figure from papers published between 2016 and 2021

^c^This figure from papers published between 2016 and 2022

^d^Neither = neither human nor animal subjects involved. The distribution of studies was 105 human, 103 neither

**Supplementary Table 2**

*Prevalence of Bias Minimisation Practices in Animal Research as Assessed by Previous Work*

| Study characteristic | | | | | Preregistration | | | Masking | | | Randomization |  | Sample size calculation | Data exclusion |
| --- | --- | --- | --- | --- | --- | --- | --- | --- | --- | --- | --- | --- | --- | --- |
| Study | Field of interest | Human or animal or both | Years of papers reviewed | Total number of paper | States preregistered (and accessible). | States not preregistered. | No statement. | Any mention | Masked outcome assessment | Masked allocation | Any mention | Group allocation | Used to calculate sample size | Any mention |
| Adewumi et al. (2021) | Addiction medicine | Both^a^ | 2014-2018 | 244 | 2.9% (2.9%) | 0% | 97.1% |  |  |  |  |  |  |  |
| Norris et al. (2021) | Addiction (Smoking) | Human | 2018-2019 | 100 | 73% (72%) |  |  |  |  |  |  |  |  |  |
| Hardwicke et al. (2020) | Social sciences | Human, neither^b^ | 2014-2017 | 156 | 0% | 0% | 100% |  |  |  |  |  |  |  |
| Bebarta et al. (2008) | Emergency medicine | Animal | 1997-2001 | 290 |  |  |  |  | 10.7% |  |  | 32.4% |  |  |
| Kousholt et al. (2022) | All (Danish) | Animal | 2009, 2018 | 500 |  |  |  |  | 23.6% vs 38% |  |  | 24%, 40.8%^c^ | 2.8%, 12.8% | 20.4%, 38.4% |
| Leung et al. (2018)^a^ | Animal welfare, analgesia or anaesthesia | Animal | 2009, 2015 | 236 |  |  |  | 19.2% vs 34.5%^c,f^ |  |  |  | 13.3%, 15.3%^c^ | 2.5%, 9.7%^c^ |  |
| Fergusson et al. (2019) | Anaesthesiology, anaesthesia & analgesia, anaesthesia, British Journal of Anaesthesia | Animal | 2008-2009, 2014-2016 | 604 |  |  |  |  | 37%, 45% |  |  | 50%, 63%^c^ | 16%, 29%^e^ | 30%, 37%^e^ |
| Hirst et al. (2014) | Biomedical | Animal | 1992-2012 | 31 systematic reviews |  |  |  |  | 35% | 15% |  | 29% |  |  |
| Ting et al. (2015) | Rheumatology | Animal | 2012 | 41 |  |  |  |  | 29% |  | 17.1%^g^ |  | 0% | 19.5%^d^ |
| Vesterinen et al. (2010) | Multiple sclerosis | Animal | 1961-2008 | 1117 |  |  |  |  | 16% |  |  | 9% | <1% |  |
| Macleod et al. (2015) | 8 Biomedical disease models | Animal | 1992-2011 | 2671 |  |  |  |  | 29.5% |  |  | 24.8% | 0.7% |  |
| Menke et al. (2020) | Biomedical | Animal | 2018 | 51 312 |  |  |  | 12.3%^f^ |  |  | 36.3% |  | 7.3% |  |

Note. The results taken from Leung and colleagues’ (2018) paper are from calculations we have done to average percentages over supporting vs non-supporting journals in order to get the percentages per year (2009 vs 2015). The original data can be found in the Supplementary table 2 of Leung and colleagues’ (2018) paper.

^a^The distribution of studies was 225 human and 19 animal.

^b^Neither = neither human nor animal subjects involved. The distribution of studies was 105 human, 103 neither.

^c^Only included studies where variable relevant

^d^This percentage describes reported attrition

^e^There is ambiguity about how this variable was coded. Despite efforts to contact the authors, it remains unclear. As such, we do not use this statistic in any comparisons

^f^This variable included justifications for not implementing masking

^g^Ting and colleagues (2015) coded a paper as having used randomization if it was included in reference to the study design (ARRIVE 2010 items 6b and c) or group allocation (ARRIVE 2010 items 11a and b (see their paper for this version of the ARRIVE guidelines. DOI: 10.1111/1756-185X.12699). While this is not identical to our operationalisation, because it was similarly broad, we have compared our results to theirs in our final analysis).

**Supplementary Table 3**

*Percentage Agreement Between Coders*

| Variable | Coder 1 & Coder 2 | Coder 1 & Coder 3 | Coder 1 & Coder 4 | All coders |
| --- | --- | --- | --- | --- |
| % agreement | 87.5 | 93.8 | 99.54 | 93.6 |

**Supplementary Table 4**

*List of All Journals in Our Dataset*

|  | **Journal name** | **Number of articles in journal** | **Percentage** |
| --- | --- | --- | --- |
| **1** | Addiction Biology | 19 | 7.45 |
| **2** | Neuropharmacology | 18 | 7.06 |
| **3** | Psychopharmacology | 11 | 4.31 |
| **4** | Pharmacology, Biochemistry and Behavior | 11 | 4.31 |
| **5** | Neuropsychopharmacology | 11 | 4.31 |
| **6** | Behavioural Brain Research | 10 | 3.92 |
| **7** | Frontiers in Pharmacology | 9 | 3.53 |
| **8** | Neuroscience Letters | 8 | 3.14 |
| **9** | Drug and Alcohol Dependence | 7 | 2.75 |
| **10** | Molecular Psychiatry | 6 | 2.35 |
| **11** | International Journal of Molecular Sciences | 6 | 2.35 |
| **12** | Behavioural Pharmacology | 6 | 2.35 |
| **13** | Progress in Neuro-Psychopharmacology and Biological Psychiatry | 5 | 1.96 |
| **14** | Journal of Psychopharmacology | 5 | 1.96 |
| **15** | Journal of Pharmacology and Experimental Therapeutics | 5 | 1.96 |
| **16** | Frontiers in Molecular Neuroscience | 5 | 1.96 |
| **17** | Pain | 4 | 1.57 |
| **18** | Journal of Neuroscience | 4 | 1.57 |
| **19** | International Journal of Neuropsychopharmacology | 4 | 1.57 |
| **20** | Frontiers in Behavioral Neuroscience | 4 | 1.57 |
| **21** | Acta Pharmacologica Sinica | 4 | 1.57 |
| **22** | Translational Psychiatry | 3 | 1.18 |
| **23** | Frontiers in Neuroscience | 3 | 1.18 |
| **24** | Behavioral Neuroscience | 3 | 1.18 |
| **25** | Thai Journal of Pharmaceutical Sciences | 2 | 0.78 |
| **26** | Proceedings of the National Academy of Sciences USA | 2 | 0.78 |
| **27** | NeuroReport | 2 | 0.78 |
| **28** | Naunyn-Schmiedeberg's Archives of Pharmacology | 2 | 0.78 |
| **29** | Molecular Medicine Reports | 2 | 0.78 |
| **30** | Journal of Neuroscience Research | 2 | 0.78 |
| **31** | Frontiers in Cellular Neuroscience | 2 | 0.78 |
| **32** | Experimental and Clinical Psychopharmacology | 2 | 0.78 |
| **33** | European Journal of Pharmacology | 2 | 0.78 |
| **34** | eNeuro | 2 | 0.78 |
| **35** | Cellular and Molecular Neurobiology | 2 | 0.78 |
| **36** | Brain Research Bulletin | 2 | 0.78 |
| **37** | Biochemical and Biophysical Research Communications | 2 | 0.78 |
| **38** | American Journal of Drug and Alcohol Abuse | 2 | 0.78 |
| **39** | ACS Chemical Neuroscience | 2 | 0.78 |
| **40** | Scientific Reports | 1 | 0.39 |
| **41** | Psychoneuroendocrinology | 1 | 0.39 |
| **42** | Phytomedicine | 1 | 0.39 |
| **43** | Physiology and Behavior | 1 | 0.39 |
| **44** | Physiological Research | 1 | 0.39 |
| **45** | Pharmacological Reports | 1 | 0.39 |
| **46** | Pharmaceutics | 1 | 0.39 |
| **47** | Pharmaceuticals | 1 | 0.39 |
| **48** | Pharmaceutical Research | 1 | 0.39 |
| **49** | Pflügers Archiv: European Journal of Physiology | 1 | 0.39 |
| **50** | Peptides | 1 | 0.39 |
| **51** | Nutrients | 1 | 0.39 |
| **52** | Nicotine & Tobacco Research | 1 | 0.39 |
| **53** | Neurotoxicology and Teratology | 1 | 0.39 |
| **54** | Neuroscience | 1 | 0.39 |
| **55** | Neurochemistry International | 1 | 0.39 |
| **56** | Neurochemical Research | 1 | 0.39 |
| **57** | Neurobiology of Stress | 1 | 0.39 |
| **58** | Neurobiology of Pain | 1 | 0.39 |
| **59** | Nature Protocols | 1 | 0.39 |
| **60** | Nature | 1 | 0.39 |
| **61** | Molecules | 1 | 0.39 |
| **62** | Molecular Pain | 1 | 0.39 |
| **63** | Metabolic Brain Disease | 1 | 0.39 |
| **64** | Learning & Memory | 1 | 0.39 |
| **65** | Journal of Venomous Animals and Toxins Including Tropical Diseases | 1 | 0.39 |
| **66** | Journal of Trace Elements in Medicine and Biology | 1 | 0.39 |
| **67** | Journal of the Experimental Analysis of Behavior | 1 | 0.39 |
| **68** | Journal of Psychiatry and Neuroscience | 1 | 0.39 |
| **69** | Journal of Pain | 1 | 0.39 |
| **70** | Journal of Neuroscience Methods | 1 | 0.39 |
| **71** | Journal of Neurochemistry | 1 | 0.39 |
| **72** | Journal of integrative neuroscience | 1 | 0.39 |
| **73** | Journal of Clinical Investigation | 1 | 0.39 |
| **74** | Journal of Biological Chemistry | 1 | 0.39 |
| **75** | International Journal of Medical Sciences | 1 | 0.39 |
| **76** | IBRO Neuroscience Reports | 1 | 0.39 |
| **77** | Human Vaccines and Immunotherapeutics | 1 | 0.39 |
| **78** | Hippocampus | 1 | 0.39 |
| **79** | Heliyon | 1 | 0.39 |
| **80** | Genes, Brain & Behavior | 1 | 0.39 |
| **81** | Frontiers in Synaptic Neuroscience | 1 | 0.39 |
| **82** | Experimental Neurology | 1 | 0.39 |
| **83** | European Neuropsychopharmacology | 1 | 0.39 |
| **84** | European Journal of Neuroscience | 1 | 0.39 |
| **85** | eLife | 1 | 0.39 |
| **86** | Drug Research | 1 | 0.39 |
| **87** | Clinical and Experimental Pharmacology and Physiology | 1 | 0.39 |
| **88** | British Journal of Pharmacology | 1 | 0.39 |
| **89** | Brain, Behavior, and Immunity | 1 | 0.39 |
| **90** | Brain Research | 1 | 0.39 |
| **91** | Biomedicine and Pharmacotherapy | 1 | 0.39 |
| **92** | Biological Psychiatry | 1 | 0.39 |
| **93** | Asian Journal of Psychiatry | 1 | 0.39 |
|  | Grand Total | 255 | 100.00 |

**Supplementary Table 5**

*Prevalence of Transparency and Replication Measures from the Current Study*

| Study Characteristic | | Results (n) | Results |
| --- | --- | --- | --- |
| Replication | Original. | 255 | 100% |
|  | Replication. | 0 | 0% |
|  | Unsure. | 0 | 0% |
| Data availability | States available and accessible. | 8 | 3.1% |
|  | States available but link broken. | 1 | 0.4% |
|  | States available but link absent. | 5 | 2% |
|  | States available upon request. | 60 | 23.5% |
|  | States not available. | 0 | 0% |
|  | No statement. | 181 | 71% |
| Code availability | States available. | 0 | 0% |
|  | States available upon request. | 2 | .8% |
|  | No statement. | 253 | 99.2% |
| Supplementary information | Yes. | 112 | 43.9% |
|  | Yes, but link absent or broken. | 13 | 5.1% |
|  | No. | 130 | 51% |
| Total |  | 255 | 100% |

*Note.* The supplementary information variable served to remind coders to check supplementary files and was not a variable of interest. It is reported here for completeness of results.

**Supplementary Table 6**

*Results of Bias Minimization Variables from the Current Study*

| Study Characteristic | | Results (n) | Results |
| --- | --- | --- | --- |
| Preregistration | States preregistered and accessible. | 0 | 0% |
|  | States preregistered but inaccessible. | 0 | 0% |
|  | States not preregistered. | 1 | .4% |
|  | No statement. | 254 | 99.6% |
|  | Registered report. | 0 | 0% |
| Masking | Yes, mentioned. | 92 | 36.1% |
|  | Statement of no masking. | 4 | 1.6% |
|  | No mention. | 159 | 62.4% |
| Randomization | Yes, any mention. | 124 | 48.6% |
|  | Other allocation method mentioned. | 17 | 6.7% |
|  | Statement of no randomization. | 2 | .8% |
|  | No mention. | 112 | 43.9% |
| Sample size justification | Power analysis/sample size calculation. | 12 | 4.7% |
|  | Past research. | 2 | .8% |
|  | No justification. | 241 | 94.5% |
| Data exclusion | Animal or data excluded. | 80 | 31.4% |
|  | Statement of no exclusion. | 8 | 3.1% |
|  | No statement. | 167 | 65.5% |
| ARRIVE | Statement of compliance. | 19 | 7.5% |
|  | Stated compliance and reported on all ‘Essential” study aspects (masking, randomization, sample size calculation, data exclusion) | 1 | .4% |
|  | Other reporting guidelines followed.^a^ | 0 | 0% |
|  | No statement of reporting guidelines. | 236 | 92.5% |
| Total |  | 255 | 100% |

*Note*. Percentages may not total 100 due to rounding.

^a^Original coding reported 52.7% of articles followed other guidelines. However, upon researching the guidelines recorded, none were found to include details on reporting of *in vivo* animal studies. As such, only this table displays the result for reporting guidelines only. See <https://osf.io/jq4c3> for other (non-reporting) guidelines mentioned.

**Supplementary Table 7**

*Results Breakdown for Articles Including Sample Size Calculations*

| Study Characteristic | | Results (n) | Results |
| --- | --- | --- | --- |
| Reason for effect size used in sample size calculation | Past research. | 4 | 33.3% |
|  | No reason provided. | 8 | 66.3% |
| Effect size type | Not mentioned. | 12 | 100% |
|  |  |  |  |
| Effect size used in sample size calculation | 0.5 | 1 | 8.3% |
|  | 0.5-0.9 | 1 | 8.3% |
|  | Unclear.^a^ | 1 | 8.3% |
|  | Not mentioned. | 9 | 75% |
| Total |  | 12 | 100% |

*Note.* This table presents a further breakdown of the results from articles that used sample size calculations (power calculations or other sample size planning techniques) to calculate the study’s sample size. Percentages may not total 100 due to rounding.

^a^This study only discussed the effect size in terms of a “25% reduction from previous papers”.

**Supplementary Table 8**

*Results Breakdown for Articles Including Mention of Data Exclusion*

| Study Characteristic | | Results (n) | Results |
| --- | --- | --- | --- |
| Exclusion reasons | Outlier. | 9 | 11.3% |
|  | Outlier and other reason. | 7 | 8.7% |
|  | No reason given. | 1 | 1.3% |
|  | Other. | 63 | 78.8% |
| Total |  | 80 | 100% |

*Note.* This table presents a further breakdown of the results from articles that mentioned exclusion of data or animals (N=80). Percentages may not total 100 due to rounding.
